# Sensorimotor cortex as a critical component of an 'extended' mirror neuron system: Does it solve the development, correspondence, and control problems in mirroring?

**DOI:** 10.1186/1744-9081-4-47

**Published:** 2008-10-18

**Authors:** Jaime A Pineda

**Affiliations:** 1 Departments of Cognitive Science and Neuroscience, University of California, San Diego, La Jolla, CA 92037-0515, USA

## Abstract

A core assumption of how humans understand and infer the intentions and beliefs of others is the existence of a functional self-other distinction. At least two neural systems have been proposed to manage such a critical distinction. One system, part of the classic motor system, is specialized for the preparation and execution of motor actions that are self realized and voluntary, while the other appears primarily involved in capturing and understanding the actions of non-self or others. The latter system, of which the mirror neuron system is part, is the canonical action 'resonance' system in the brain that has evolved to share many of the same circuits involved in motor control. Mirroring or 'shared circuit systems' are assumed to be involved in resonating, imitating, and/or simulating the actions of others. A number of researchers have proposed that shared representations of motor actions may form a foundational cornerstone for higher order social processes, such as motor learning, action understanding, imitation, perspective taking, understanding facial emotions, and empathy. However, mirroring systems that evolve from the classic motor system present at least three problems: a development, a correspondence, and a control problem. Developmentally, the question is how does a mirroring system arise? How do humans acquire the ability to simulate through mapping observed onto executed actions? Are mirror neurons innate and therefore genetically programmed? To what extent is learning necessary? In terms of the correspondence problem, the question is how does the observer agent know what the observed agent's resonance activation pattern is? How does the matching of motor activation patterns occur? Finally, in terms of the control problem, the issue is how to efficiently control a mirroring system when it is turned on automatically through observation? Or, as others have stated the problem more succinctly: "Why don't we imitate all the time?" In this review, we argue from an anatomical, physiological, modeling, and functional perspectives that a critical component of the human mirror neuron system is sensorimotor cortex. Not only are sensorimotor transformations necessary for computing the patterns of muscle activation and kinematics during action observation but they provide potential answers to the development, correspondence and control problems.

## Background

Human beings are social creatures to the extent that interactions with members of their own species, and especially the ability to understand and infer the intentions and beliefs of others, has become of predominant importance in their daily life. Whether for cooperation or non-cooperation, a core assumption of this viewpoint is that such social interactions spring from a distinction between self and others. It can be argued that at least two hierarchically-organized, overlapping and interacting neural systems have evolved and developed to manage self-other distinctions and hence social interactions [1]. One system, part of the classic motor system, is more specialized for the preparation and execution of motor actions that are self realized and voluntary, while the other appears to be more involved in capturing and understanding, at a basic and involuntary level, the actions of non-self or others. For our purposes, actions are defined as sequences of movements that together solve a motor problem [2] and that involve at least four levels of behavioral complexity: intention, kinematics, goal-object identity, and the physical consequences of the action [1]. Motor preparation and execution circuitry includes, among others, the premotor cortex, supplementary motor area, sensorimotor cortices, and parts of the inferior parietal cortex. The second system, of which the mirror neuron system (MNS) is part, has been described as the canonical action 'resonance' system in the brain – one that has evolved to utilize or share many of the same circuits involved in motor control [3]. Mirroring or 'shared circuit' systems are assumed to be important for resonating, imitating, and/or simulating the actions of others. Although no consensus exists, a number of researchers have proposed that shared representations of motor actions, or the action understanding properties of this system, may form a foundational cornerstone for higher order social processes, including motor learning, action understanding, imitation, perspective taking, understanding facial emotions, and empathy [4-8]. This means that adopting someone else's viewpoint or perspective at the very least requires that the other's actions be *understood*; else no accurate prediction of their behavior can be made.

However, a mirroring system that evolves and is adapted from the classic motor system presents at least three major problems: a development, a correspondence, and a control problem. In terms of the development problem the question is whether humans acquire the ability to mirror by mapping observed onto executed actions? That is, how exactly does a mirroring system arise? Are mirror neurons innate and therefore genetically programmed? Is learning necessary? And, what role does sensorimotor cortex play? A number of studies have indicated that imitation of facial and hand gestures in both human and non-human primates suggest the existence of mirroring systems in infancy [9-11]. Likewise, electroencephalography (EEG) and near infrared spectroscopy studies in humans show sensitivity to executed versus observed actions, as well as between live and televised actions [12-16] suggesting the existence of mirroring as early as 6–7 months of life. However, none of these studies directly answers the development questions posed. On the other hand, computational models of mirroring activity propose that sensorimotor transformations, via Hebbian learning, can in fact mediate such development.

In terms of the correspondence problem the question is how does the observer agent determine what the observed agent's activation pattern is in order to match it? Or, as Brass and Heyes [17] stated the problem with respect to imitation, "When we observe another person moving we do not see the muscle activation underlying their movement but rather the external consequences of that activation. So, how does the observer's motor system 'know' which muscle activations will lead to the observed movement?" Resonance becomes particularly difficult when the observer and observed do not share the same embodiment and affordances, that is, they do not share all "action possibilities" latent in the environment. One partial solution to this problem, of course, exists in the implicit nature of a mirroring system, i.e., a system that *evokes motor representations by movement observation*. That is, if motor actions already exist as part of the observer agent's movement repertoire then observation of action, even when partially triggered, can be sufficient to evoke the representation. This solution clearly makes sensorimotor transformations, as part of a mirroring system, necessary for solving such a correspondence problem.

Finally, in terms of the control problem, the issue arises because an efficient mirroring system ought to be turned on only when needed. However, it has been shown repeatedly that activation of internal motor representations via observation occurs automatically. Neuroimaging studies, for example, show that simple passive observation is enough to generate motor activation. The question then is how to control a system for efficiency when it is turned on automatically? Or, as others have stated the problem succinctly: "Why don't we imitate all the time?" The existence of neural inhibitory and monitoring mechanisms as partial solutions to this control problem has been acknowledged [3], although the specific anatomical implementation of such mechanisms is unknown. Brass and colleagues [18], for example, found that the fronto-median cortex and the right temporo-parietal junction were activated when an instructed movement had to be executed during observation of an incongruent movement. The implication being that high level areas are involved in inhibition of imitative response tendencies. Another solution centers on phasic changes in oscillatory EEG activity as inhibitory control mechanisms. This is consistent with the role of sensorimotor cortex as a critical region for mirroring based on its common output path role in motor and simulation-based representations. More specifically, we hypothesize that oscillatory activity, such as mu rhythms in sensorimotor cortex, play a key role in controlling mirroring processes.

**Figure 1:**
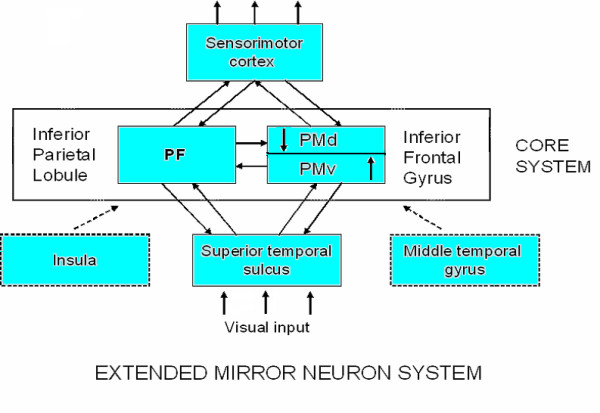
**Schematic of areas in the human brain that contain mirror neurons (inferior parietal lobule and inferior frontal gyrus) and make up the 'core'system.** The 'extended' mirror neuron system involves additional brain areas, e.g., insula, middle temporal gyrus, and somatosensory cortex, which connect to the core system and perform transformations on the data critical for mirroring and simulation.

Mirroring activity can be conceptualized as occurring in a gradient. At one end of the spectrum, the mimicry of another individual's postures, facial expressions, vocalizations, movements and mannerisms is often executed in the absence of awareness, as occurs in the chameleon effect, motor empathy, motor contagion, or emotional contagion [19]. At the other end of the spectrum, it has been suggested that simulation based on mapping of observed actions onto one's own motor system necessitates the interaction with semantic/cognitive circuits for conscious action understanding to occur [20,21]. We conceptualize this spectrum of action understanding as reflecting four levels of behavioral complexity, i.e., intentions, goals, patterns of muscle activation, and kinematics, as has been suggested by Hamilton and Grafton [1]. Furthermore, we argue that these levels of processing can be mapped onto differences in activation in different components within a 'core' and an 'extended' mirror neuron system (see Figure 1). Although it remains to be definitively shown, differential activation of the various components of this mirroring system most likely result as a function of the task, working memory, motivational and/or attentional factors involved. In this paper, we argue from an anatomical, physiological, modeling, and functional perspectives that one critical component of an 'extended' mirror neuron system is sensorimotor cortex. This region is necessary not only for computing the patterns of muscle activation and kinematics during action observation but provides potential answers to the development, correspondence and control problems in mirroring.

### The 'core' MNS

**Figure 2:**
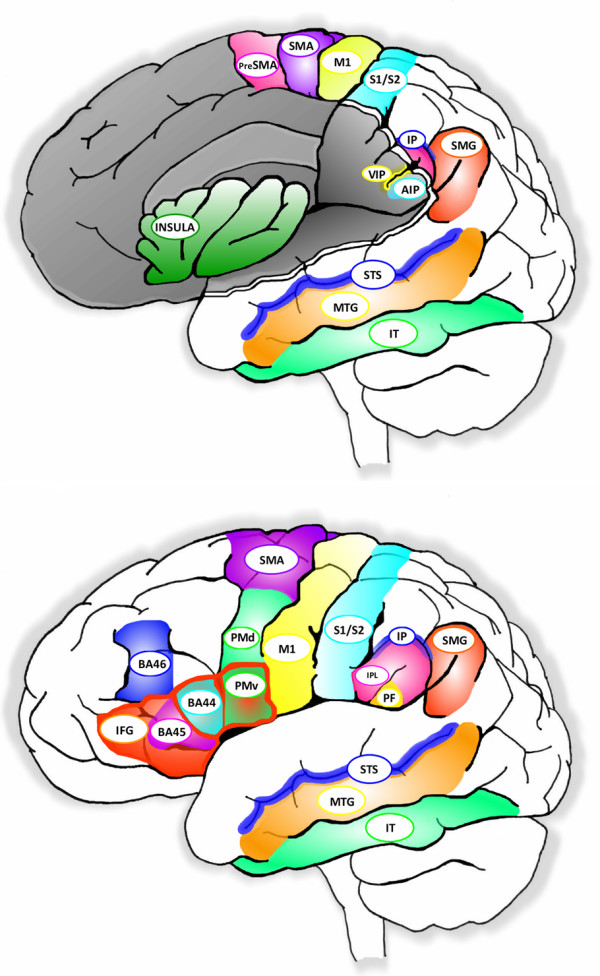
Anatomical view of a human brain showing areas involved with the mirror neuron system.

**Figure 3:**
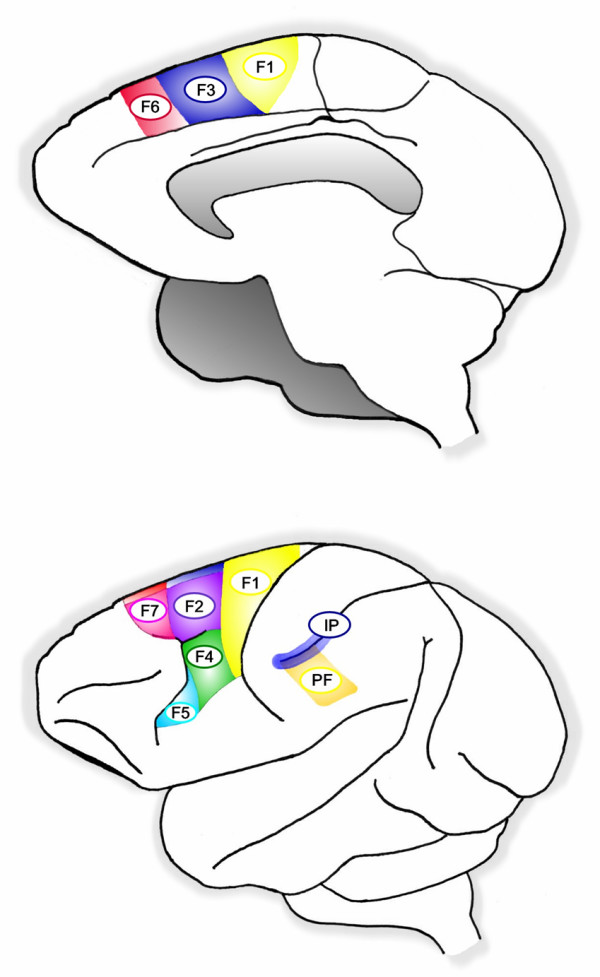
Anatomical view of a macaque monkey brain showing areas involved with the mirror neuron system.

The mirror neuron system has been widely defined as consisting of three interrelated areas: ventral premotor area (PMv) of the inferior frontal gyrus (area F5 in monkeys), parietal frontal (PF) in the rostral cortical convexity of the inferior parietal lobule (IPL), and the superior temporal sulcus (STS) (see Figures 1, 2 and 3, as well as Table 1 for a description of these areas). The mirror neuron circuit in monkeys [4,22] begins in the rostral part of the superior temporal sulcus, although no mirror neurons *per se *have been reported in this area. Information is then thought to flow to the parietal frontal area on the rostral cortical convexity of the inferior parietal lobule. A subset of the cells in this region has mirror properties: i.e., they discharge both when the monkey executes as well as observes an action. Parietal frontal area, in turn, sends projections to area F5 of the ventral premotor area, where a subset of cells (10–20%) exhibits mirror properties. Thus, the core mirror neuron system would be defined as those areas that contain mirror-like neurons, which at this point includes primarily the rostral convexity of the inferior parietal lobule or parietal frontal area and ventral premotor area.

**Table 1:** Abbreviations and functional descriptions of anatomical areas

**Abbreviation**	**Name**	**Function**
AIP	Anterior intraparietal	visually guided grasping; comparable to monkey area F5

BA44	Brodmann's area 44	Broca's area; language production

BA46	Brodmann's area 46	rostral portion of the IFG; sustained attention and working memory

F2	Monkey area F2	integrates body position and motor acts

bF4	Monkey area F4	codes for peripersonal space; caudal part of PMv

F5	Monkey area F5	codes for distal movements; rostral part of PMv

F6	Monkey area F6	pre-SMA; learning of new motor sequences

IFG	Inferior frontal gyrus	action observation and imitation

Insula	Insular cortex	body representation and subjective emotional experience

IP	Intraparietal sulcus	guidance of limb and eye movement

IPL	Inferior parietal lobule	post-central sulcus/anterior border, intraparietal sulcus/superior border, and the lateral fissure/anterior inferior border.

IT	Inferotemporal cortex	identification and categorization of objects

M1	Primary motor cortex	patterns of muscle activation

MTG	Middle temporal gyrus	subserves language and semantic memory processing, visual perception, and multimodal sensory integration

PF	Parietal frontal	rostral convexity of IPL

PMd	Dorsal premotor	simultaneous encoding of multiple movement

PMv	Ventral premotor	monkey area F5; analogous to BA 44; pars opercularis of IFG

S1	Primary somatosensory	kinematics

S2	Secondary somatosensory	integrating across body parts; frontoparietal operculum and lateral convexity of IPL

SMA	Supplementary motor	planning motor actions

SMG	Supramarginal gyrus	spatial orientation and semantic representation

STS	Superior temporal sulcus	visual information entry area

VIP	Ventral intraparietal	comparable to monkey area F4

Single unit studies in the premotor cortex of macaque monkeys indicate that neurons in area F5, particularly in the caudal portion of the inferior frontal gyrus (IFG), are indistinguishable from neighboring neurons in terms of their motor properties and discharge in response to executed and observed actions [23] (for a review see [4]). The implication is that when a monkey observes an action, particularly one that is in its motor repertoire, a subset of neurons in this region 'mirrors' the activity and represents the motor action in its own premotor cortex, revealing a type of observation/execution matching system. This type of observation/execution activity has been shown to be selective for goal-directed, meaningful actions supporting the idea that actions are organized with respect to distal goals [24]. More recently, another subpopulation of neurons in the same area of the monkey has been found that discharges both when the animal performs a specific action as well as when it sees or hears the same action performed by another individual [25,26]. That is, these cells represent in an individual's motor cortex not only the execution of an action (motor representation) but also the 'observation' of that action performed by others (visual representation), as well as its auditory correlates (auditory representation). In other words, auditory mirror neurons allow for a mapping of specific heard actions onto the motor programs for executing the same actions.

Individual human mirror neurons cannot be studied directly except under unusual circumstances [27]. Nonetheless, the evidence suggests that the motor related part of Broca's region is located in the caudal portion of the inferior frontal cortex, in what is Brodmann's area 44, and there appears to be a homology between area F5 in the monkey and area 44 in humans. Area 44 is involved in interfacing external information about biological motion and internal motor representation of hand/arm and mouth actions [28,29]. Hence, the existence of an analogous mirroring system in the homologous human brain regions has been supported by indirect population-level measures such as electroencephalography [12,30-34], magnetoencephalography [35], transcranial magnetic stimulation [36], positron emission tomography [37,38] and functional magnetic resonance imaging [19,39,40]. Fadiga and colleagues [36], for example, found that motor evoked potentials over motor cortex were enhanced in response to transcranial magnetic stimulation when subjects observed another individual performing an action relative to when they detected the dimming of a light. Iacoboni and colleagues [39] measured blood flow in Brodmann's area 44 and found increases during the observation and performance of actions. Other studies have reported activations with similar properties in the parietal cortex [40,41], as well as the superior temporal sulcus [42,43]. In general, the human mirror neuron system appears active during the performance and observation of the same action and is hypothesized to be necessary for imitative learning [44], comprehending the actions of others [24,45], understanding the goal of another's actions [46], interpreting facial expressions [19,47], and exhibiting empathy [19].

### The 'extended' MNS

It has been shown that we activate our own motor, somatosensory, and nociceptive representations while perceiving the actions of others, while at the same time activating representations of our own emotional states as well as facial expressions while witnessing others' emotions [48]. At minimum, this activation of shared representations for action and emotion requires a variety of anatomical and functional circuits that together might be called the 'extended' mirror neuron system. Undoubtedly, the core mirror neuron areas, as described previously (see Figure 1), are anatomically connected with many other regions that contribute significantly to the subsequent elaboration of the information [49,50]. Those regions may themselves not contain mirror neurons *per se*, such as the superior temporal sulcus, but the level of transformation performed on the data would make them critical to the outcome and part of an extended mirroring process.

The arguments as to why the superior temporal sulcus, despite the lack of mirror neurons, is considered part of a mirror neuron system are both anatomical and functional [51]. It is an area that contains neurons that respond to biologically relevant actions of the head, body, and eyes, as well as to static pictures that merely imply biological motion [52]. Furthermore, this area is reciprocally connected to the parietal frontal area in the inferior parietal lobule. However, the functional significance of the mirror neuron system has to be understood in its connections to many other neural systems [20]. Thus, the degree to which brain areas in these other systems play a critical role in action understanding or in any of the processes attributed to the core mirror neuron system would define their inclusion as part of an extended circuit.

The extant evidence supports inclusion of a number of areas into an extended definition of the mirror neuron system. For example, the subjective sense of how one feels is theorized to be based upon anterior insula representations of the body. This is assumed to provide a foundation for emotions and perhaps even for self-awareness that could allow for simulation of future actions, in order to use the feelings generated by the simulation to guide decision making [53]. Singer and colleagues [54] found, in a functional magnetic resonance imaging study, that empathy for pain involves simulating the unpleasant, aversive qualities of the pain (the motivational significance of pain) but not its precise somatic characteristics. In another study, Saarela and Hari [55] used photos of facial expressions from chronic pain sufferers which varied in the intensity of depicted suffering. Not only were bilateral anterior insula, left anterior cingulate, and left inferior parietal lobe activated, but the amount of these activations correlated with subjects' estimates of the intensity of observed pain. Clearly, the insula has an important role in mirroring and should be considered part of the extended mirroring system. Likewise, observation-evoked motor activity, as well as mirror-type activity, has been reported in dorsal premotor cortices [56,57], while the middle temporal gyrus (MTG) and adjacent superior temporal sulcus are often found to show augmented blood-oxygen level dependent (BOLD) responses during action execution and action observation [58,59]. Finally, and most relevant to the argument in this paper, primary and secondary motor and somatosensory cortices often contain voxels active during both action execution and observation/listening [13,58,60,61].

## Anatomical perspective

Sensorimotor cortex has been implicated in determining the organization and representation of conceptual knowledge of concrete objects and actions [47,62,63]. Behavioral and functional magnetic resonance imaging studies support the notion of mental representations grounded in sensorimotor interactions with the real world [64,65]. Such representations are most likely involved in understanding and producing actions and emotions of conspecifics via simulation of observed behavior [24,47,66,67]. In order to understand the role of sensorimotor cortex in mirroring, simulation and in understanding the actions of others, as well as to understand how sensorimotor cortex solves the development, correspondence and control problems, it is helpful to understand its anatomical and functional properties, and more precisely the underlying computations necessary for movement and movement understanding.

### M1 connections

A number of neurophysiological [13,68-70] and neuroimaging [71-73] studies have shown that mirror-like activity occurs in several brain regions including the human primary motor (M1) and somatosensory (S1) cortices. Why should these sensorimotor cortices be active during action observation? Since the majority of studies have examined hand movements, sensorimotor activation may simply be a side effect of the strong reciprocal connections between premotor cortex and sensorimotor areas. Premotor cortex is typically subdivided into dorsal (PMd) and ventral (PMv) regions (see Figure 2). Stepniewska et al. [74,75], Greenlee et al., [76] and Dum and Strick [77] have shown that the densest inputs from premotor areas to the orofacial and digit representation in primary motor cortex originate from dorsal and ventral premotor areas. The ventral premotor area connects with the digit and orofacial portions of primary motor cortex and also has extensive connections with somatosensory areas (S1, S2, 3a). Dorsal premotor area also connects with proximal forelimb and trunk areas of primary motor cortex [75] and is connected directly to spinal cord [78].

Dum and Strick [77] performed tracer studies in the Cebus monkey and employed a number of techniques, including electrophysiologically mapping the digit representations, to check against their tracer results, and used dual tracers to compare multiple inputs to the primary motor cortex in the same animal. The results showed that for digit representations, primary motor cortex receives the strongest input from the ventral and dorsal premotor areas. These areas in turn receive their strongest reciprocal input from primary motor cortex, and it appears that the same area in motor cortex projects to both ventral and dorsal premotor areas. Furthermore, there is also a strong amount of interconnection between the ventral and dorsal premotor areas as well. The argument made by Dum and Strick [77] is that such areas form a densely interconnected network concerned with the generation and control of hand movements. Hence, primary motor cortex is active because premotor areas are active. However, Kilner and Frith [51] offer an alternative explanation to this passive response activation. They suggest that premotor and primary motor areas code executed action in different coordinate systems. Premotor areas code targeted action primarily in an extrinsic reference framework that *encodes the kinematic aspects of the action*, that is, target and hand are defined relative to each other in space. In contrast, primary motor neurons code the same action based on an *intrinsic framework of muscles and joint space that is related to the shaping of hand and digits*. Therefore, understanding actions and inferring intentions require both the premotor areas for a kinematic description and primary motor cortex for a description of the patterns of muscle activity necessary to execute the action.

In a recent study examining single-cell properties of primary motor cortex and dorsal premotor area neurons, Tkach et al. [79] identified a set of cells that exhibited observation- and execution-based activation, a major characteristic of mirror neurons. However, their study did not show whether these cells also responded to the interaction between subject and target object, a characteristic of mirror neurons. In another study, Stefan et al. [80] showed that primary motor cortex displays mirror-like activity in response to movement observation, is capable of forming motor memories, and is involved in motor learning. In their study, transcranial magnetic stimulation was used to show that observation of another individual performing simple repetitive thumb movements gives rise to a kinematically specific memory trace of the observed motions in this motor region.

### S1 connections

In one of the first functional magnetic resonance imaging studies to examine 'tactile empathy,' Keysers et al. [81] showed that secondary somatosensory area (S2), in the fronto-parietal operculum, extending onto the lateral convexity of the inferior parietal lobule and presumably involved in integrating information across body parts, is activated both when the participants were touched and when they observed someone or something else getting touched by objects. This area receives somatosensory, visual, and polysensory inputs from primary somatosensory cortex, and extrastriate visual areas, as well as from areas in the posterior parietal lobe, suggesting that it may be involved in integrating somatosensory information with other sensory modalities [82]. Furthermore, this secondary somatosensory area has extensive reciprocal connections with ventral premotor areas, as well as with prefrontal cortex (Brodmann's area 46). Curiously, Keysers et al. [81] did not show primary somatosensory cortex activation to the observation of touch. In contrast, in a more recent study [83], it was reported that the primary somatosensory cortex was indeed activated in non-synesthesia subjects by the mere observation of touch and that this activation was somatotopically organized. Furthermore, the mirror neuron system in these subjects (including the premotor cortex, superior temporal sulcus, and parietal cortex) was activated by the observation of touch to another human more than to an object. Interestingly, in a synesthesia subject these areas appeared to be overactive, i.e., above the threshold for conscious tactile perception.

It has also been suggested that somatosensory representations are critical for processing emotion [84]. Adolphs et al. [85] provided a theoretical framework in which they suggested that recognizing emotion in another person engages both visual representations of the perceptual properties of facial expressions and somatosensory representations of the emotion that may simulate how one would feel if making the shown facial expression. Specifically, they found that lesions in the right somatosensory cortex, as well as in anterior supramarginal gyrus and to a lesser extent in the insula, were associated with impaired recognition of emotions from human facial expressions. Individuals having only somatosensory lesions showed impairment. They also reported a significant correlation between impaired somatic sensation and impaired recognition, but only in the right hemisphere and not shown in relation to motor impairments. A study by Hagen et al. [86] showed that posterior inferior frontal gyrus receives extensive projections from secondary somatosensory areas and responds to somatosensory stimulation. Inferior frontal gyrus also has projections from primary somatosensory cortex, area 7b, and the ventral frontal opercular region [82]. Monkey studies have suggested that functionally this region may be important for working memory for tactile stimuli [87].

## Computational perspective

One computational view of mirror neuron functionality places it in the context of auto-associative networks whose links are strengthened via Hebbian synaptic plasticity. In this view, neurons become capable of sharing representations primarily through an associative learning mechanism. That is, these auto-associative or content addressable memory architectures are established when an agent acts. That is, associations naturally occur among the motor, somatosensory, vestibular, auditory, visual, and other inputs when a movement is executed. It is hypothesized that linking the observation of movement (visual input) to extant motor representations such that later observed actions can retrieve these stored patterns automatically can explain how the mirror neuron system develops. This notion of associational learning is supported by recent evidence showing that it is possible to manipulate the selectivity of the human mirror system, and thereby make it operate as a countermirror system, by giving participants training to perform one action while observing another [88]. These results by Catmur and colleagues strongly argue that mirroring is not entirely innate [9] nor unchangeable once the patterns are learned; but most likely develop through sensorimotor associational learning [89,90] as a product and a process of social interaction.

This idea is also supported by neuroimaging studies that purport to show that mirror neuron activity varies as a function of the observer's expertise. Calvo-Merino et al. [91] showed that ballet and capoeira dancers observing actions they were trained to perform showed greater activity in premotor and parietal areas. Similarly, Haslinger et al. [92] showed similar effects for piano players observing piano playing. It's also been shown that familiarity (which presumably involves enhanced sensorimotor activation) activates premotor cortex more than non-familiar actions [33].

Oztop and Arbib [49,50] have argued that mirroring properties are an exaptation of a more basic neuronal function, namely that of providing feedback for visually-guided grasping movements. Although the evolution of how such self-hand movements relate to objects to recognize the manual action of others is unclear, Fagg and Arbib [93] have suggested, based on various computational models, that dorsal stream information flowing through the anterior intraparietal area is where the grasps *afforded *by the object (i.e., those actions that are made possible by the object) are extracted, while area F5 selects and drives the execution of the grasp. Prefrontal cortex, which receives object recognition information from inferotemporal cortex (IT), biases F5 selection to choose the appropriate possible actions for the task. Furthermore, a variety of prefrontal areas, such as F6 (pre-SMA), Brodmann's area 46 (dorsolateral prefrontal cortex), and F2 (dorsal premotor cortex) are proposed to be involved in biasing F5 to respond to task constraints, working memory, and instruction stimuli, respectively. Once the location of the object is known, the information flows to the motor programming area F4, which computes the reach. The information about the reach and the grasp is fed into primary motor cortex to control the hand and arm.

Although this computational framework of how actions are organized with respect to distal goals is incomplete, there is agreement that primary motor cortex computes muscle activations given reach targets and limb postures in the presence of noise [94,95]. Other computational perspectives argue that mirroring systems involved in recognizing actions can be understood within a predictive coding framework, or more formally, as equivalent to Bayesian inference within a hierarchical structure [96]. This refers to a computational framework for inferring the causes (intentions, goals, and motor commands) of sensory inputs (observed kinematics) by minimizing prediction error at all levels of a cortical hierarchy. Indeed, the notion that muscle activity is a linear projection of primary motor cortex output has been called into question [97-99]. Rather, primary motor cortex receives input from ventral premotor area, which appears to code object locations in a hand-centered frame of reference. It then sends its output to muscles via the spinal cord. This sensorimotor common output path for both motor and mirroring-based representations makes primary motor and primary somatosensory areas strategic for inhibitory control and monitoring mechanisms.

## Physiological perspective

### Mu rhythms

Although no mirror-type neurons (except see [79]) have been reported in sensorimotor cortex, of particular importance is that studies using electroencephalography and magnetoencephalography have indicated that power in mu rhythm oscillations in this region, including alpha (8–13 Hz) and beta (14–25 Hz) components, is modulated by the observation and imagination of movement in the same way that self movement produces such modulation [100,101]. It has been known since the early 1950s that planning and execution of movement, especially of the hand, produces desynchronization or suppression of this rhythm [102,103], while inhibition of motor behavior enhances it or produces synchronization in animals [104]. This has led to a taxonomy of mu rhythm properties [103]. Hari et al. [13] were the first to show an involvement of primary motor cortex in the human mirror neuron system by showing modulation of the beta (20 Hz) component of the mu rhythm during the observation of hand actions. They have provided extensive magnetoencephalography evidence that primary motor cortex is activated both during the observation and execution of motor tasks [105,106].

### Mu rhythm properties

Using high-density, whole-head magnetoencephalography recordings and surface Laplacian transformations, a number of studies have shown that the alpha and beta mu oscillations have their origin in sensorimotor cortex [107]. However, the sources of the beta component appear to be more anterior to those of the alpha component, which originate in postcentral somatosensory cortex [108]. Indeed, significant negative correlations between both 10-Hz and 20-Hz mu rhythms and blood-oxygen level dependent signals have been reported in frontal and parietal cortices [109,110]. Caetano et al. [107] indicated that the modulation of the alpha rhythm lasted approximately 600 ms longer during action versus observation or listening conditions. They attribute this to a proprioceptive feedback signal during self movement and proposed that such a signal may enable the mirroring system to attribute agency to the correct source. It is also the case that the difference in coding action in distinct coordinate systems proposed by Kilner and Frith [43] maps well onto the alpha and beta components of mu oscillation.

Furthermore, recent studies have shown that synchronized mu rhythms in the hand area of motor cortex produces desynchronized mu rhythms in the foot or tongue area [101,111] suggesting a lateral inhibitory network in sensorimotor regions. Furthermore, the differential reactivity of the mu oscillations to different contingencies suggests the existence of distinct bands: one that is somatotopically non-specific (8–10 Hz), one that is somatotopically specific (10–13 Hz) [112], and one (14–30 Hz) that may reflect corticomuscular processes [113,114].

### Relationship to mirroring

Until recently, mirror neurons had not been directly reported in sensorimotor cortex creating a problem relating the changes in mu rhythms to activity in the mirror neuron system. One explanation for the functional similarities was that sensorimotor activity involved a downstream modulation, via cortico-cortical connections, from premotor areas, including inferior frontal gyrus [12]. As was argued previously, the inferior frontal gyrus and sensorimotor cortex are reciprocally interconnected. However, this raises a potential problem. If sensorimotor cortex is activated by premotor commands during the observation of actions, which are similar to the motor commands generated during the behavior itself, then how is it possible to differentiate between the two and avoid movement when we observe actions? The most intuitive explanation is that the motor activity we observe is being actively gated by upstream and downstream areas. Indeed, we would argue that changes in mu rhythm reflect such signal gating. Hummel et al. [115] have shown that a significant increase in 11–13 Hz oscillations over sensorimotor cortex occurs during inhibitory control of a memory trace, while during retrieval of the trace there was a decrease in such oscillations. Results from other human electroencephalographic studies suggest that an increase in power in the beta range is associated with inhibition of the excitatory state of the motor cortex [116]. There is also clinical evidence regarding the origin of this inhibition in patients with frontal lobe damage that exhibit 'unwilled' automatic movements [117]. These clinical studies suggest that the prefrontal, anterior cingulate, and supplementary motor cortices may contribute the necessary inhibition to prevent triggering of movement commands realized in activated motor and premotor cortical areas.

Measuring cortico-spinal excitability by using transcranial magnetic stimulation during action observation has proven to be an excellent way to explore how neural networks are involved in the mirror neuron system and hence in social cognition. These studies have shown that the observation of action affects motor corticospinal [36,118], intracortical [119], or spinal excitability [120]. Furthermore, such stimulation appears to desynchronize rhythms in the primary motor cortex [13,60,121] strongly suggesting that mirror neurons from ventral premotor cortex modulate activity in primary motor cortex.

## Functional perspective

Numerous electroencephalography, magnetoencephalography, and transcranial magnetic stimulation studies have shown that changes in mu rhythm oscillations during both execution and observation of actions reflects mirroring properties. Mu suppression has been observed during the observation of moving hands compared to the observation of bouncing balls [30], point-light biological movements [32,122], complex social interactions [31], and familiar versus unfamiliar actions [33] indicating that mu rhythms in humans are not only sensitive to object-directed movement but to general biological motion having social significance. Mu rhythm suppression is typically greater during the execution of object-directed hand movement compared to simple hand movement. Likewise, it is greater during object-directed hand movement observation than in simple hand position observation [123,124]. These phenomenological properties resemble what has been reported for monkey mirror neurons. Both respond to execution and observation of object-directed movement [23], as well as cognitive imagery. Their overlapping neural sources in sensorimotor frontoparietal networks further support the argument that they are related and involved in linking perception to action, which may be a critical component in the development of higher level cognition.

Although mirror neurons are primarily thought to be involved in perception and understanding of motor actions [4], they may also play a critical role in higher order cognitive processes such as imitation [44,50,125], theory of mind [7,47,126], language [50,127,128] and empathy [129]. A number of studies performed over the past several decades suggest that children and adults with autism spectrum disorder suffer from impairments that closely parallel the functioning of the mirror neuron system [130-133]. Indeed, the DSM-IV diagnostic criteria for autism spectrum disorders include deficits in social and communicative skills such as imitation, empathy, and shared attention, as well as restricted interests and repetitive patterns of behaviors. Elucidating their neuroetiology has been a challenge because behavioral manifestations vary both in severity as well as expression, such as Autism (low-, medium, high-functioning), Asperger's Disorder, or pervasive developmental disorder – not otherwise specified or PDD-NOS [134,135]. To date, no single explanation has been able to account for the broad and varied profile of these deficits [136]. However, a recent convergence of evidence on autism spectrum disorders has implicated the mirror neuron system. In fact, Williams et al. [137,138] suggested that early failures of this system could result in the cascade of developmental impairments seen in autism.

Though recognized over 50 years ago, the cause of imitation impairments in autism has yet to be identified, but several hypotheses about its origin have been proposed. One hypothesis suggests that this is a core deficit that could impede early affective, social and communicative development [139]. Specifically, it is suggested that imitation deficits result from an inability to form and coordinate social representations of self and others via amodal or cross-modal representation processes – the type of function ascribed to mirror neurons. Neuroimaging and neurophysiological studies support this argument [30,133,140]. However, the hypothesis has been challenged recently, especially the existence of mirroring-based imitation deficits [141,142].

Nonetheless, the discovery of mirror neurons provides a testable basis for some of the major deficits seen in autism spectrum disorders. These specialized cells show increased firing rates not only during execution of an action (motor representation) but also during 'observation' of the corresponding action performed by others (visual representation) [4]. The mirror neuron system thus appears capable of directly mimicking the action it perceives, or performing a simulation of the action without accompanying motor execution. This type of observation/execution matching system is hypothesized to provide a mechanism for translating seeing into doing, an ability that may be especially critical for imitation learning but also for the development of empathy, and theory of mind. Therefore, a number of strands of convergent evidence provide the rationale for a non-invasive investigation of the mirror neuron system in autism and for studying the effects of an intervention strategy centered on mirroring function. First, there is relatively direct evidence for mirror neuron system involvement in autism spectrum disorders. Second, many known impairments affect functional domains potentially associated with the mirror neuron system, such as imitation and theory of mind [137]. Third, there is increasing evidence for an electrophysiological signature of mirroring activity. Finally, activity-dependent reorganization is a neural property that can be effectively recruited for the remediation of disordered behavior.

## Conclusion

Theories of knowledge representation can be categorized by whether or not they resort to 'embodied' versus 'disembodied' explanations [143]. Embodied theories argue that conceptual content and sensorimotor content are essentially the same, whereas disembodied theories see sensorimotor explanations as necessary but not sufficient to explain action concepts [62]. Embodied theories, therefore, argue for a central role of sensorimotor transformations in the representation of conceptual knowledge and assume that *simulation *requires a reactivation of sensorimotor areas. These ideas have been put forth as motor theories of action recognition, suggesting that motor processes are involved in the recognition of visually presented actions [144]. Furthermore, it has been suggested that sensorimotor processes characterize the "...semantic content of concepts in terms of the way we function without bodies in the world" and thus are intimately involved in language, theory of mind, and conceptual processing [126].

The arguments we have made in this paper, based on anatomical, physiological, modeling, and functional perspectives, are consistent with embodied explanations. That is, sensorimotor transformations are a critical component of an extended mirroring system and necessary not only for computing the patterns of muscle activation and kinematics during action observation but for simulation and understanding. Furthermore, sensorimotor transformations and the anatomical connections of sensorimotor cortex with core and extended mirror neuron system areas provide potential answers to the development, correspondence and control problems in mirroring. Nishitani and Hari [60] have shown with magnetoencephalography that activity in primary motor cortex during action observation occurs later than inferior frontal gyrus. This suggests that sensorimotor contributions to the understanding of the actions of others may be at the output end of mirror neuron system processing. As a final output path for motor and simulation-based representations, sensorimotor cortex allows for what is perhaps the critical property of mirroring systems – evoking motor representations through the observation of movement. Thus, sensorimotor cortex offers a solution to some of the more serious problems posed by mirroring systems because it offers a common output path for motor control and simulation-based transformations. These transformations can also become the foundational cornerstone for higher order social processes, such as motor learning, action understanding, imitation, perspective taking, understanding facial emotions, and empathy [4,5]. Furthermore, they help connect the neurophysiology of mu rhythms to the process of mirroring.

Until recently, the sensorimotor cortex has not been considered part of a mirroring system primarily because no evidence existed that neurons in these regions responded to the passive observation of actions. However, a number of studies reviewed above [13,79,145] have provided support for the idea that 'mirror-like' properties occur in sensorimotor neurons to the observation of actions, including changes in mean firing rate, sensitivity to preferred direction and to the presence of a target, as well as oscillatory power modulation in specific frequency bands, raising the prospect that these areas are indeed an integral and necessary part of an extended mirroring system.

Sensorimotor learning, presumably mediated through Hebbian synaptic plasticity and auto-associational mechanisms, appears to answer the questions regarding the development of the mirror neuron system. This clearly suggests that the mirror neuron system is neither entirely innate nor inflexible and in fact may dynamically adjust to changing inputs. This gives some basis to the notion that dysfunctional mirroring systems, such as have been reported in children and adults with autism spectrum disorders, may be susceptible to therapeutic improvement with the right type of input [33,146]. Our own conceptual model of how mirroring develops has been particularly influenced by the work of Kilner et al. [96], which can be described as a probabilistic matching mechanism. These authors argue that one problem in inferring the cause or an intention of an action is that the problem is ill-posed "because identical movements can be made when performing different actions with different goals." The mirror neuron system and other such systems solve this problem, it is argued, by the use of predictive coding on the basis of Bayesian inference. This means that the likely cause of an observed action is inferred by minimizing the prediction error at all levels of the hierarchy involved during action-observation. This type of model assumes that the areas involved in action understanding are arranged hierarchically and that the connections between them are reciprocal. The developmental time course of such wiring quite likely determines the types of mirroring processes that come online, from mimicry to functional context-sensitivities during action observation.

Solutions to the correspondence problem have required the existence of general representations of the body that are shared between observer and observed agent. The discovery of mirroring systems is consistent with that solution. That is, automatic activation of *existing *motor representations in sensorimotor cortex constrains the body representation mapping that occurs between observer and observed agents even when these agents do not share the same embodiment and affordances, i.e., all "action possibilities" latent in the environment [17]. This means that the system takes advantage of internal rather than external observation and thus imitation or learning occur from actions made by oneself or made by another on oneself [147].

Finally, the solution to the control problem in mirroring is grounded in the final common path architecture of sensorimotor cortex for both motor and simulation-based representations. This allows for shared access to inhibitory control circuits. To that end, changes in oscillatory activity in the mu band appear to reflect such control. Thus, the weight of the evidence suggests that sensorimotor circuits are part and parcel of the two hierarchically-organized, overlapping and interacting neural systems that have evolved and developed to manage self-other distinctions and hence social interactions [1].

## Competing interests

The author declares that they have no competing interests.
